# Comparative pharmacokinetics and pharmacodynamics of intravenous artelinate versus artesunate in uncomplicated *Plasmodium coatneyi*-infected rhesus monkey model

**DOI:** 10.1186/s12936-016-1456-6

**Published:** 2016-09-06

**Authors:** Paktiya Teja-Isavadharm, Duangsuda Siriyanonda, Maneerat Rasameesoraj, Amporn Limsalakpeth, Nitima Chanarat, Natthasorn Komcharoen, Peter J. Weina, David L. Saunders, Montip Gettayacamin, R. Scott Miller

**Affiliations:** 1 Department of Immunology and Medicine, Armed Forces Research Institute of Medical Sciences, 315/6 Rajvithi Road, Bangkok, 10400 Thailand; 2 Department of Veterinary Medicine, Armed Forces Research Institute of Medical Sciences, 315/6 Rajvithi Road, Bangkok, 10400 Thailand; 3 Walter Reed Army Institute of Research, 503 Robert Grant Avenue, Silver Spring, MD 20910 USA; 4 Director Research Programs, Walter Reed National Military Medical Center, Bethesda, MD 20889 USA; 5 US Army Medical Materiel Activity, Ft Detrick, Frederick, MD 21702 USA; 6 AAALAC International Southeast Asia Office, Bangpla, Bangplee, Samutprakarn 10540 Thailand; 7 Global Health Division, Bill & Melinda Gates Foundation, 250/830 Moo3, Bangpla Soi 18, Seattle, USA

**Keywords:** Monkey malaria, Artelinic, Artelinate, Artesunate, Pharmacokinetics, Pharmacodynamics, Parasite clearance

## Abstract

**Background:**

The US Army designed artelinate/lysine salt (AL) to overcome the instability of sodium artesunate in aqueous solution (AS). To select the most efficacious artemisinin treatment, direct comparison was performed in an uncomplicated non-human primate malaria model.

**Methods:**

Splenectomized rhesus monkeys were inoculated with *Plasmodium coatneyi* and on day six, single equimolar loading dose of IV AL (11.8 mg kg^−1^) or IV AS (8 mg kg^−1^) were administered followed by 1/2 the first dose once daily for 2 more days. Blood smear were performed twice daily and the number of parasites were counted microscopically. Blood samples were obtained after the first dose within 6 h for pharmacokinetic (PK) and ex vivo pharmacodynamic evaluation by simultaneously measuring plasma drug concentration and anti-malarial activity against *Plasmodium falciparum* in vitro.

**Results:**

The anti-*P. coatneyi* in vivo activity of both compounds were comparable, but the ex vivo anti-*P. falciparum* potency of the IV AS regimen as administered was sevenfold higher than that of IV AL. Comparing in vivo pharmacodynamics of AL and AS, daily assessed parasite counts showed comparable 99 % parasite clearance times (PC99: 2.03, 1.84 day), parasite clearance rates (5.34, 4.13 per min) and clearance half-life (PCt_1/2_: 7.79, 10.1 h). This study showed strong and significant inverse correlation between PCt_1/2_ and t_1/2_ of AS + DHA, and AUC_0–∞_ of DHA, and correlated with V_z_ of AS (r^2^ > 0.7, p ≤ 0.002). Lastly, following IV AL, there was a modest inverse correlation between PCt_1/2_ and C_max_ (r^2^ 0.6, p ≤ 0.04). Although all tested monkeys recrudesced subsequently, two died following AL regimen before parasite clearance. While the aetiology of those deaths could not be definitively determined, pathologic evidence favoured a sepsis-like syndrome and suggested that severe malaria was more likely than drug toxicity.

**Conclusion:**

The model demonstrated that both AS and DHA contributed to the anti-malarial activity of IV AS, while IV AL activity was largely restricted to the parent drug. Parasite clearance was strongly and linearly dependent on drug exposure for both artemisinin regimens. However, IV AS had higher ex vivo potency against *P. falciparum*, leading to an IND filing for GMP manufactured AS in the United States.

**Electronic supplementary material:**

The online version of this article (doi:10.1186/s12936-016-1456-6) contains supplementary material, which is available to authorized users.

## Background

Despite reductions in morbidity and mortality, severe malaria still kills a half a million persons worldwide each year, including travelers [1]. Severe malaria occurs when *Plasmodium* infection is complicated by serious organ failure or metabolic abnormalities which develop rapidly and can progress to death within 24–48 h, and requires parenteral therapy. Since 1991, quinidine gluconate remains the only FDA-approved drug available for severe malaria infection in the US. It is available in limited supply, requires continuous infusion with a loading dose in an intensive care unit for cardiac and glycaemic monitoring, and resistance has been documented [2, 3]. Artesunate (AS) was developed and tested as a replacement for quinine. AS has now been receiving WHO-prequalification status since 2010 and adopted as the recommended treatment; but a Good Manufacture Practice (GMP) product remains inaccessible in many countries [4]. Intravenous AS was associated with a lower risk of hypoglycaemia and has been shown to significantly reduce the risk of death from severe malaria compared to IV quinine [5, 6]. Artesunate is also a more practical option in resource-poor settings since administration is simpler (by bolus injection) and does not require slow rate-controlled infusion as well as cardiac monitoring [5, 7].

During the testing of parenteral artesunate, the US Army, through the Walter Reed Army Institute of Research, had considered developing another artemisinin derivative, artelinic acid-l-lysine salts (AL) and was patented in 1988 [8] as anti-malarial agents for clinical treatment of severe malaria. Artelinate, a semi-synthetic derivative of artemisinin, does not convert readily to dihydroartemisinin (DHA) was designed to overcome the instability of sodium artesunate in aqueous solution, which must be prepared at the bed side before injection. Pre-clinical efficacy and toxicity studies were used to select one of these artemisinin candidates as potential treatments for severe malaria and eventual FDA registration. Comparative anti-malarial efficacy of IV AL and AS in rat malaria model showed that AL had a superior anti-malarial potency than AS in terms of parasitaemia clearance [9]. However, this evidence also showed that AL was more toxic than AS in rats [10, 11].

Several non-human primate malaria models serve as useful surrogates to evaluate new drugs prior to testing in humans. *Plasmodium coatneyi* is primate malaria that causes tertian malaria in splenectomized Asian macaques, complicated by sequestration of the infected erythrocytes and a high mortality rate in untreated animals [12]. This model most closely mimics severe and complicated malaria caused by *Plasmodium falciparum* [13, 14]. Limited efforts have used this model to study toxicity of drug due to brain-damage induced by lipid-soluble depot artemisinins [13, 15]. The present study compared the pharmacokinetics and pharmacodynamics of AL and AS in the uncomplicated *P. coatneyi* infected rhesus monkey model to test the new candidate drugs for severe malaria treatment.

## Methods

The United States Army Medical Component, Armed Forces Research Institute of Medical Sciences (USAMC-AFRIMS) Institutional Animal care and Use Committee and the Animal Use Review Division, US Army Medical Research and Materiel Command reviewed and approved the protocol. Research was conducted in 2002 in compliance with the Animal Welfare Act and other federal statutes and regulations relating to animals and experiments involving animals and adheres to principles stated in the Guide for the Care and Use of Laboratory Animals, NRC Publication, 1996 edition. The USAMC-AFRIMS animal care and use programme has been accredited by Association for Assessment and Accreditation for Laboratory Animal Care International since 1999.

### *Plasmodium coatneyi*-Rhesus monkey model

*Plasmodium coatneyi*-infected RBCs (wild-type strain originally donated by W.E. Collins, CDC, Atlanta, GA) were maintained as cryopreserved stabilates at AFRIMS, Bangkok, Thailand. The stabilate stock was tested and confirmed negative for B-virus, SRV, SIV and STV-1 to ensure no adventitious viruses transmission were introduced to the monkey colony. Twenty-two adult colony-born, Indian-origin rhesus macaques (*Macaca mulatta*) naïve monkeys were splenectomized and recovered well more than 1 month prior to entering the study. All monkeys were free from B-virus, SIV, SRV and STLV antibodies. Animal health screening included complete physical examination, CBC, ALT and creatinine. Twenty-two animals were randomized to two experiments of 11 monkeys. Each experiment included ten experimental monkeys (IV AL and AS) and one control animal. The control monkeys received intramuscular quinine treatment and the experimental animals received intravenous artemisinin candidates.

### Infected donor monkey

For each experiment, after thawing a stabilate of *P. coatneyi*-infected erythrocytes, 1 ml aliquot was inoculated intravenously to the test monkey. The monkey was sedated with ketamine HCl (5–15 mg kg^−1^, IM) for this procedure. Parasitaemia levels were monitored daily. After the parasite density reached >50,000 parasites µl^−1^, 5–20 ml of blood was drawn from a femoral vein into a heparinized tube. Blood was divided into 1 ml aliquots (about 5 × 10^6^ RBC with a 1 % parasitaemia) for inoculation of the study animals. Then, the donor animal was treated with chloroquine hydrochloride 10 mg kg^−1^ day^−1^ IM for 5–7 days. This was the standard treatment regimen for *P. coatneyi*-infected control animals at AFRIMS based on local protocols. The injectable chloroquine hydrochloride (Aralen™ 40 mg base/ml) was given by intramuscular route. The injection has been practical since it can be given with minimal animal handling using the “squeeze cage” mechanism. Animals were monitored daily and the treatment range of 5–7 days was used as the benchmark for when blood smear evaluation became negative.

### Parasitaemia and clinical monitoring

Animals were observed at least twice daily to assess clinical signs and correlates of severe malaria. Based on our previous model validation, blood smears were performed to detect malaria parasites starting prior to inoculation on day 0 and continued daily (0700–0800 h). The monkeys were physically restrained in the squeeze cage restraint apparatus and approximately 0.1–0.2 ml whole blood was collected from each monkey by lancet puncture of the lateral auricular vein. Thick and thin smears were made on the same microscope glass slide and subsequently fixed in methanol and stained with Giemsa. When the smears were positive, white blood cell count (WBC) and/or red blood cell count (RBC) were also performed using automated haematology analyzer Sysmex K800 to calculate parasite density. Haematocrits were checked as needed. At study days 6–12, additional blood samples were obtained from all animals in the afternoon (1500–1600 h) to monitor parasitaemia levels. Daily blood smears were continued until two consecutive days with negative smears. Then the smear monitoring schedule was reduced to two times weekly for 2 weeks and then weekly for 6 weeks after completion of the last treatment. Treatment failures and/or recrudescence were treated with IM quinine dihydrochloride 60 mg kg^−1^ loading dose, followed by 30 mg kg^−1^, every 8 h for 3 days.

Treatment was initiated at parasitaemias ≥3 % or >200,000 parasites µl^−1^ (on study day 6 morning) due to rapid increasing in parasitaemia levels observed in previous model development and dose-ranging studies (unpublished data). Blood smear staining and parasitaemia determination took about 2 h. The first dose of treatment was decided after the results were confirmed. The eleven monkeys were treated as one cohort when the majority met the treatment criteria and allowed for practical blood drawing and processing for PK sampling. The control monkey received quinine treatment and the ten experimental animals received intravenous artemisinin derivative candidate. Animals were pole-and-collar trained and sat in restraining chairs to receive the IV injection and phlebotomy without the use of chemical restraint to avoid potential confounding and depression by concomitant anesthetic administration.

### Chemicals

Artelinic acid (WR255663), 4-(10′ dihyro-artemisinin-oxymethyl) benzoic acid hemihydrate, was prepared as an l-lysine salt (MW 573.719) powder at Walter Reed Army Institute of Research (WRAIR; Silver Springs, MD) and shipped to AFRIMS through WRAIR Chemical Information. Artesunic acid (MW 384.425) and 5 % sodium bicarbonate solution was obtained from Guilin Pharmaceutical, Guangxi, China and imported by Atlantic Pharmaceutical Co., Ltd., Bangkok, Thailand. Artelinate/lysine salt (AL) powder prepared with 1 g of the acid content was equivalent to 1.35 g of the salt weight. Intravenous AL was prepared from a stock solution containing 40 mg L^−1^ of AL by dissolving the calculated amount of the AL salt in a solution of 0.45 % NaCl/0.1 % l-lysine (w/v), sterilized and filtered through 0.2 µm cellulose acetate. Additional IV solutions with the required concentrations of AL was made by appropriate dilution of the 40 mg L^−1^ solution with normal (0.9 %) saline containing 0.1 % l-lysine, followed by filtration through a syringe filter prior to use. Intravenous artesunate/sodium salt (AS) was prepared by dissolving the acid in 5 % sodium bicarbonate just prior to injection per manufacturer instructions.

### Dosing regimen

Single, equimolar doses 20 μmol of IV AL (11.8 mg kg^−1^) and IV AS (8 mg kg^−1^) were administered as a rapid bolus injection over a 1-min period. These doses were chosen as the minimal effective doses (from previous model development and validation studies, unpublished data) and blood was collected for pharmacokinetic analysis. After a week wash-out and recovery period, *P. coatneyi* (1 mL of >50,000 parasitized RBCs per microlitre or 5 × 10^6^ RBC with a 1 % parasitaemia) were inoculated from a donor monkey into all the monkeys. The treatment was initiated at day 6 after inoculation at a parasitaemia ≥3 % or >200,000 parasites μL^−1^ when the cohort of animals was minimally symptomatic. Drugs were given for 3 days with a loading dose initially followed by ½ of the first dose on the 2nd and 3rd days. Consequently, each monkey served as its own control for intra-subject variation, and differences are likely due to the disease effects from malaria infection.

### Blood collection and sample preparation

Blood was collected for pharmacokinetic/pharmacodynamics (PK/PD) analysis after the first dose only due to rapid clearance of artemisinin derivatives. Heparinized blood was collected at 0, 5, 20, 40 min, 1, 3, and 6 h post-dose and plasma samples were separated within 2 h by centrifugation (1500*g*, 10 min). Aliquots of samples were made for simultaneous measurement by HPLC [16] and bioassay [14], and stored at −80 °C until analysis. All glassware was silanized before use with a 0.5 % aqueous solution (v/v) of Pierce Aquasil (Rockford, IL, USA) according to the manufacturer’s recommended procedure. Solid-phase extraction was performed on C-18 elution cartridge (3.0 ml, J.T. Baker Co.) using a VAC ELUTE SPS24 sample processing station (Analytichem International, Harbour City, CA, USA). The cartridge was preconditioned by sequential washing with 2.5 ml acetonitrile, 2.5 ml methanol and 2.5 ml water. One ml of distilled water was added to the cartridge before adding plasma sample (0.5 ml). Then, the cartridge was washed with 2.5 ml distilled water, 2.5 ml of 0.05 % H_3_PO_4_ (pH 4.5) and 2.5 ml of 20 % acetonitrile and completely dried by closing the vacuum for 15 min. The analytes were collected by eluting with 0.5 ml methanol three times and then with 0.5 ml 90:10 *n*-butyl chloride: ethyl acetate twice. The organic phases were pooled. The combined organic phases were then evaporated to dryness under a stream of nitrogen at room temperature. Extraction residues were reconstituted with 50:50 ethanol: water (0.3 ml), at least 16 h before injection. The injection volume was 100 µl for all samples.

The plasma concentration of parent drugs, AL and AS, and their primary metabolites, DHA and 2-hydroxyartelinate (OHAL) respectively, were measured by HPLC with electrochemical detection in reductive mode (HPLC-ECD) [16], which was the only method for artemisinin compounds then (Additional files 1 and 2). The total anti-malarial activity of the drug and all active metabolite(s) in plasma were measured by bioassay against in vitro *P. falciparum* clone (W2) and expressed as DHA equivalents [14] (Additional file 3).

### Chromatography

HPLC with reductive electrochemical detection was performed using a model BAS 200B liquid chromatography (Bioanalytical Systems, Inc., West Lafayette, Indiana, USA). A Nova-Pak^®^ C_18_ (3.9 × 150 mm, Waters Corporation, Milford, MA, USA) was used with 40 % acetonitrile in sodium acetate buffer (pH 3.6) as mobile phase. Detection was performed using a thin dual-layer glassy carbon electrode run in parallel mode at a potential of −1.0 V versus Ag/AgCl. The system was operated at the following temperatures: mobile phase 35 °C, column and detector oven 30 °C, cell 31 °C with the flow rate of 1.5 mL min^−1^. Artemisinin (ART) was used as an internal standard. Samples were injected via Gilson model 231 XL sampling injector equipped with a 100 µL loop, a Gilson model 401 dilutor/pipettor, a sampler controller keypad (Gilson Medical Electronics, Middleton, WI, USA), and a Valco electrically actuated switching valve (Valco Instrument Co. Inc) was used. This system automatically deoxygenated and injected sample, which permits the high sample throughput associated with automated injection. Data were acquired and analysed using a Millennium^32^ Chromatography Manager (Waters Corporation, Milford, MA, USA).

Standard curve and quality control samples were prepared by spiking plasma with 50:50 ethanol:water solutions of AS with DHA or AL with OHAL. The final concentration range was from 10 to 2000 ng mL^−1^ plasma. Artemisinin 250 ng mL^−1^ was added as an internal standard to all samples.

### Ex vivo *Plasmodium falciparum*-based bioassay (BA)

The plasma anti-malarial activities of the artemisinin derivatives were measured using a previously published method [14]. In brief, plasma was incubated with protein A to remove the majority of immunoglobulin (IgG) that results in the lysis of the parasites in the erythrocytes (heat treatment was not used due to the effect on the stability of artemisinin derivatives in plasma). After centrifugation, the supernatant (100 µL) was transferred to a 96-well flat bottom plate. Twofold serial dilutions were then made using heat inactivated pooled control serum (human:rhesus = 1:1). A suspension of malaria (W2 clone) infected erythrocytes was added to each well. The microtitre plate was placed into a gas-tight plexiglass chamber, flushed with a gas mixture of 90 % CO_2_, 5 % O_2_, and 5 % N_2_, and placed into an incubator (37 °C). Following 24 h incubation, the plate was removed from the chamber and pulsed with [^3^H]-hypoxanthine solution to each well and incubated for another 18–20 h before harvesting. The incorporation of [^3^H]-hypoxanthine by the parasites in each well was determined by counting in a scintillation counter.

A dose–response curve for each plasma sample was constructed by plotting [^3^H]-hypoxanthine uptake on the Y-axis against the corresponding dilution factor on the X-axis after logarithmic transformation. The dilution factor value that inhibits parasite growth by 50 % (DF_50_) of each plasma sample was determined. Using the DF_50_ value, the anti-malarial activity of the plasma sample was extrapolated from a linear standard curve using DHA, the most potent artemisinin derivative, and expressed as DHA equivalents.

### Assessment of parasite counts

Blood smear were collected once daily in the morning. Once positive parasitaemia was observed and reached 2000 parasites μL^−1^, blood smears were collected twice, in the morning and afternoon. Blood for slides preparation were collected from ear for thick and thin smear preparation. Blood films on glass slides were air dried, fixed with 100 % methanol, stained with Giemsa, and read under ×100 magnification oil emersion for parasite determination.

First, a thick smear was performed for parasite detection by reading the slides at least 50 fields under ×100 magnification oil emersion. If no parasite was detected in all 50 fields, “negative” parasitaemia was reported. If on average five parasites were detected/field under ×100 magnification oil emersion, the number of parasitized RBCs was counted per 100 WBC. This number was used for the number of parasites μL^−1^ of blood using the formula:

$$ {\text{Parasites }}\upmu {\text{L}}^{{{ - }1}} \,{\text{blood}} = \left[ {{{{\text{number}}\,{\text{of}}\,{\text{counted}}\,{\text{parasites}}} \mathord{\left/ {\vphantom {{{\text{number}}\,{\text{of}}\,{\text{counted}}\,{\text{parasites}}} {100\left( {{\text{WBC}}_{\text{S}} } \right)}}} \right. \kern-0pt} {100\left( {{\text{WBC}}_{\text{S}} } \right)}}} \right] \times {\text{WBC}}\,{\text{count}}\,\upmu {\text{L}}^{{{ - }1}} \,{\text{blood}} $$

Blood CBC was determined by automated haematology analyzer Sysmex K800 every other day. When parasite density was greater than ten parasites/field, parasites were counted per 1000 RBC. Thin smear was used when number of parasite was greater than five parasites per field in thick film. For counting parasite density/RBC, a grid micrometre was used and the number of parasitized RBCs was used for calculation. If the parasite density was less than ten parasites μL^−1^ the determination of parasitaemia was counted per 100 WBC (calculation method of parasite number was the same as thick smear).

The percentage of infected RBC was determined by enumerating the number of infected RBCs in relation to the number of uninfected RBCs. One thousand RBCs were counted and calculated as follows:

$$ \% \,{\text{infected RBCs}} = \left( {{{{\text{No}} .\,\,{\text{infected}}\,{\text{RBCs}}} \mathord{\left/ {\vphantom {{{\text{No}} .\,\,{\text{infected}}\,{\text{RBCs}}} {{\text{Total}}\,{\text{No}} .\,{\text{RBCs}}\,{\text{counted}}}}} \right. \kern-0pt} {{\text{Total}}\,{\text{No}} .\,{\text{RBCs}}\,{\text{counted}}}}} \right) \times 100 $$

The number of asexual parasites μL^−1^ of whole blood was determined by counting white blood cells (WBC) in high-power fields containing a total of 500 parasites if the ratio of parasites/WBC was more than one, or the number of parasites per 1000 WBCs was counted if the ratio of parasites/WBC was less than one. The parasitaemia level was calculated as the product of the parasite/WBC ratio and the WBC count (microscope filed that is well-populated with WBCs (20 WBCs/field) and five views were used for parasitaemia count).

### In vivo pharmacodynamics (iPD) analysis

The parasite density-time curve was constructed and the parasite clearance parameters were estimated using Parasite Clearance Estimator [17]. The parameters were parasite clearance rate (PCR), clearance half-life (PCt_½_), PC50, PC95, PC99 (PCn—defined as the time from initiation of treatment to the time parasites were cleared by n %). In addition, the parasite clearance time (PCT) by conventional method (defined as the time from initiation of treatment to the time the first two consecutive negative blood smears) was assessed by microscopy.

### Pharmacokinetic analysis

Non-compartmental analysis was used to derive pharmacokinetic parameters of AS and its metabolite DHA, AL and its metabolite OHAL, using WinNonlin™ Standard Edition, v 2.1 (Pharsight Corporation, Cary, NC, USA). The PK parameters determined were the elimination rate constant (λ_z_) was calculated by least-squares regression analysis of the ln-linear portion of the plasma concentration–time curve (the number of time points >3). The elimination half-life (t_1/2_) was calculated from the ratio of 0.693/λ_z_, the maximum concentration (C_max_) in plasma and the time to reach C_max_ after dosing (T_max_) were obtained by inspection, the area under the concentration–time curve (from time zero to 20 min, AUC_0–20_, and zero to infinity, AUC_0–∞_) was estimated by the linear trapezoidal rule. The apparent total body clearance (Cl) and volume of distribution (V_z_) associated with the terminal phase were calculated as dose/AUC, and Cl/λ_z_, respectively.

### Statistical analysis

Statistical comparison between dosage regimens was performed using the Mann–Whitney U test and all the values reported were in median with 25th and 75th interquartile. The correlation between two variables was performed using simple linear regression model with least square method. Results were considered statistically significant when the p value was <0.05. (Prism 6 for Windows v. 6.01)

## Results

### Clinical results and in vivo pharmacodynamics (iPD)

**Fig. 1:**
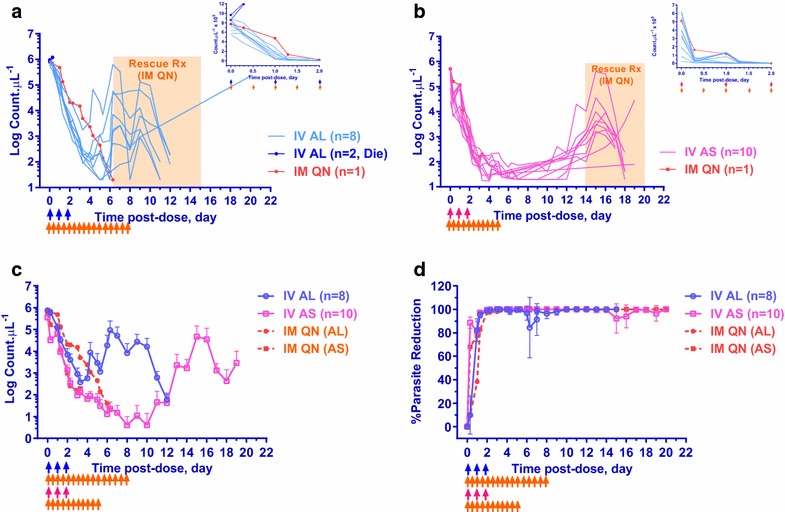
Parasite density-time profiles. Parasite density-time profiles of all test monkeys receiving IV AL (*blue*), IV AS (*pink*) and control monkeys receiving IM QN (*orange*) with corresponding *color coded arrows* indicating dosing time: **a** IV AL 11.8 mg kg^−1^ showing eight test monkeys (*light blue*) and two monkeys that eventually died within 6 h after the first dose (*dark blue*) and **b** IV AS 8.0 mg kg^−1^, in log scale and linear scale in *inset*. **c** Mean + 95 % confidence bar in plasma of *P. coatneyi* infected rhesus monkeys (*solid line*) including quinine control (*dotted line*) for each treatment. **d** Percentage (mean ± 95 % CI) of parasite reduction after treatment

Monkeys in the two treatment groups had comparable age and weight (Table 1). The parasite density of each control monkey receiving quinine were 773,550 and 510,260 parasites μL^−1^ for AL and AS group, respectively, which were within the range of the corresponding experimental monkeys. Parasite clearance-time profiles were shown in Fig. 1a–c (Additional file 4). Although animals treated with IV AL had statistically significant higher starting parasite density (727,850 vs 261,452; p 0.0005) and longer PC95 (1.22 vs 0.85 days, p 0.01), there were not significantly different in the PC99 (2.03 vs 1.84 day, p 0.3), PCR (5.34 vs 4.13 count.min^−1^, p 0.1), and PCt_1/2_ (7.79 vs 10.1 h, p 0.1). Both formulations cleared the parasites so rapidly (Fig. 1d) that there were not sufficient data points to determine PC50 for all animals, only 4 of 8 for IV AL and 3 of 10 for IV AS with their corresponding PC50 values varying from 2.72–10.9 h and 0.32–5.88 h respectively. Unexpectedly after IV AL injection, there were two deaths in the infected animals within 2.5 h. The blood smear from both monkeys taken at time of death had comparable parasite densities of 1.2 × 10^6^ parasites μL^−1^ with a percentage of parasitized red blood cells at 15 and 20 %. There was evidence of malaria and hepatic centrilobular degeneration consistent with shock. Though no clear etiology of death was determined, the findings favoured a diagnosis of severe malaria over drug toxicity, particularly since these doses were well tolerated by the other animals in the experiment. Six animals failed treatment because parasite counts never reached zero and PCT could be determined in only two animals at 4.0 and 5.3 days. In contrast, PCT could be determined in all monkeys in the IV AS group with a median of 7.0 days and IQR between 5.1 and 8.0 days. However, the two monkeys with the most rapid PCT occurred at 4.0 and 5.0 days. Median PCT of the remaining eight monkeys was 8.0 days with an IQR between 5.8 and 8.3 days. There was no relationship between PCT and initial parasite density. Furthermore, the parasites in the monkeys treated with IV AL, on average, kept increasing on day 4 after dosing while those treated with IV AS continued to decrease and cleared by 1 week post-treatment (Fig. 1c). However, parasites recrudesced in all monkeys at a median (range, n) of 13 (9–19, 10) days in the IV AS group and 6.5 (6–7, 2) days in the IV AL group.

**Table 1:** The initial status of the monkeys and in vivo pharmacodynamics, parasites clearance values

Parameters	Unit	Rx	Median	25th IQT	75th IQT	p^a^
Monkey age	Year	IV AL	8	8	10	1.0
		IV AS	9	7	12	
Monkey weight	kg	IV AL	8.4	6.7	9.0	0.8
		IV AS	7.7	6.9	8.7	
Parasite density^b^	Count mL^−1^	IV AL	727,850	639,946	848,916	<0.001
		IV AS	261,452	139,771	489,064	
Parasites/erythrocyte (PE)	%	IV AL	12.0	11.3	12.7	<0.001
		IV AS	6.60	4.23	6.83	
Parasites clearance rate (PCR)	Count min^−1^	IV AL	5.34	4.32	6.54	0.1
		IV AS	4.13	3.69	5.28	
Parasites clearance half-life (PCt_1/2_)	Hr	IV AL	7.79	6.36	9.63	0.1
		IV AS	10.1	7.88	11.3	
95 % Parasites clearance (PC95)	Day	IV AL	1.22	1.13	1.27	0.01
		IV AS	0.847	0.557	1.03	
99 % Parasites clearance (PC99)	Day	IV AL	2.03	1.89	2.08	0.3
		IV AS	1.84	1.47	2.01	
Parasites clearance (PCT)^c^	Day	IV AL	None	None	None	NA
		IV AS	7.00	5.07	8.00	

^a^Mann Whitney test

^b^Geometric mean and 95 % confidence interval

^c^Conventional method

### Pharmacokinetics (PK)

**Fig. 2:**
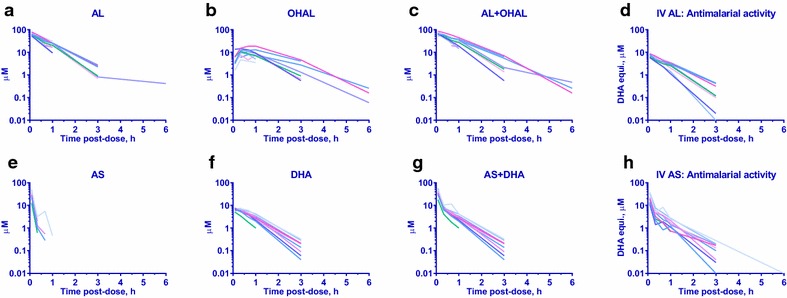
Spaghetti plots of drug concentration–time profiles. Spaghetti plots of IV AL 11.8 mg kg^−1^ (**a**–**d**) and IV AS 8.0 mg kg^−1^ (**e**–**h**) in plasma of *P. coatneyi* infected rhesus monkeys showing drug concentration–time profiles of parent drugs (AL, AS), their metabolites (OHAL, DHA), combined parent + metabolite (AL + OHAL, AS + DHA), and their corresponding anti-malarial activities-time profiles against *P. falciparum* W2 clone

**Fig. 3:**
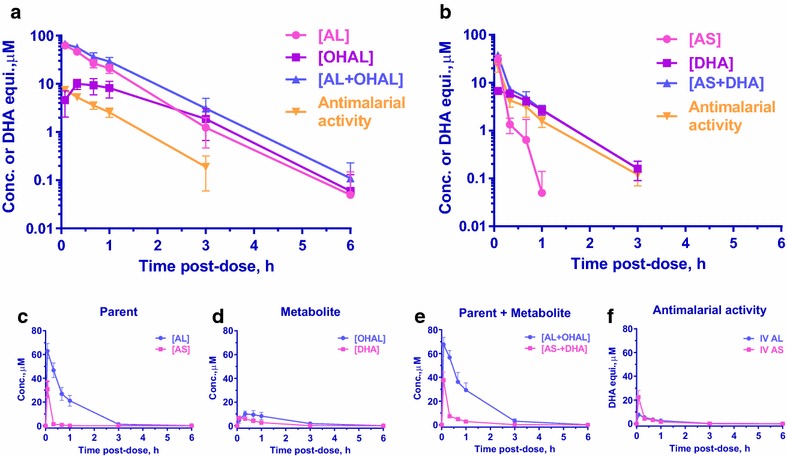
Mean drug concentration and anti—*P. falciparum* activity-time profiles. Drug concentration–time profiles (PK) measured by HPLC-ECD and anti-malarial activities-time profiles (ePD) simultaneously measured by ex vivo bioassay against *P. falciparum* W2 clone following equimolar 20 µmol kg^−1^ dose of **a** IV AL 11.8 mg kg^−1^, n = 8 and **b** IV AS 8.0 mg kg^−1^, n = 10 [reproduced from Fig. 5c (14)] in plasma of *P. coatneyi* infected rhesus monkeys. *Top panel* showed the parent drugs, their metabolites, combined parent and its metabolite, and anti-malarial activities of each parenteral regimen. *Bottom panel* showed direct comparison of **c** parent drugs (AL vs AS), **d** their metabolites (OHAL vs DHA), **e** combined parent + metabolite (AL + OHAL vs AS + DHA), and **f** their anti-malarial activities (IV AL vs IV AS). Values are mean ± 95 % CI

The concentration–time profiles for each drug, its metabolite and anti-malarial activities for each animal are shown in Fig. 2 with mean values and 95 % CI shown in Fig. 3. Comparison of PK profiles for IV AL and IV AS showed no major differences between pre-infection and malaria infected states (Additional files 5, 6, 7, and 8). As a result, the PK and effect kinetics of ex vivo anti-malarial activity profiles or ex vivo pharmacodynamics (ePD) of the two IV candidates in infected monkeys were directly compared. All PK and ePD parameter estimates were derived from Fig. 2.

Table 2 shows PK parameter estimates of IV AL compared to IV AS. Almost all PK variables for IV AL were significantly greater than that of IV AS (p < 0.0001). For parent drug AL, C_max_ was twofold (60.2 vs 28.2 μM), t_1/2_ was eightfold (28.2 vs 3.47 min), AUC_0–∞_ was 13-fold (3706 vs 288 μM.min), and AUC_0–20_ was 3-fold (925 vs 288 μM min) greater than that of AS. With the exception of the percentage of AUC_0–20_ compared to total AUC (%AUC_0–20_), AL was 1/4 that of AS (24.6 vs 97.0 %). Moreover, the clearance (Cl) and the volume of distribution (V_z_) of AS was 13-fold (72.3 vs 5.60 L kg^−1^ min^−1^) and 1.5-fold (0.397 vs 0.259 L kg^−1^) greater than that of AL. The primary metabolites of AL and AS detected were OHAL and DHA respectively. The half-life values of OHAL (42.2 min) and DHA (31.1 min) were 1.5 and 9.0 fold longer than their parent drugs. In addition, the AUC_0–∞_ of OHAL was only 0.33 of its parent AL while that of DHA was 1.58 fold greater than its parent AS.

**Table 2:** Pharmacokinetics of intravenous artelinate and artesunate in plasma from *P. coatneyi* infected rhesus monkeys

Pharmacokinetics		Rx	Parent compound (P)				Metabolite (M)				M/P	% AUC_0–20_	
Parameter	Unit		Median	25th IQT	75th IQT	p^a^	Median	25th IQT	75th IQT	p^a^		AUC_0–∞_	
C_max_^b^	mM	IV AL	60.2	55.9	65.3	<0.0001	10.9	9.10	13.4	<0.0001	0.18		
		IV AS	28.2	24.3	38.0		7.23	6.52	7.41		0.26		
t_1/2_^c^	min	IV AL	28.2	25.8	35.4	<0.0001	42.2	33.2	47.6	0.02	1.50		
		IV AS	3.47	3.09	5.15		31.1	25.0	34.5		8.97		
AUC_0–∞_^d^	mM min	IV AL	3706	2845	4218	<0.0001	1260	944	1519	<0.0001	0.33		
		IV AS	288	260	399		453	415	494		1.58		
AUC_0–20_^e^	mM min	IV AL	925	849	1028	<0.0001	117	100	153	0.7		24.6	
		IV AS	288	253	394		116	107	122			97.0	
Cl^f^	L kg^−1^ min^−1^	IV AL	5.60	4.85	7.88	<0.0001							
		IV AS	72.3	52.4	79.9								
V_z_^g^	L kg^−1^	IV AL	0.259	0.241	0.294	0.07							
		IV AS	0.397	0.311	0.507								

^a^Mann Whitney Test

^b^ *C*_*max*_ maximum concentration of drug reached in plasma

^c^ *t*_*1/2*_ elimination half-life

^d^ *AUC*_*0–∞*_ area under the concentration–time curve from zero to infinity

^e^ *AUC*_*0–20*_ area under the concentration–time curve from zero to 20 min

^f^ *Cl* apparent total body clearance

^g^ *V*_*z*_ volume of distribution

### Ex vivo pharmacodynamic (ePD)

The effect kinetic parameters of ex vivo anti-malarial activity (measured by BA expressed as DHA equivalents), or ePD parameters of IV AL compared to IV AS were shown in Table 3. The anti-malarial activity t_1/2_ values were essentially the same at 30 min. The AUC_0–∞_ was not significantly different between groups (426 μM.min for IV AL and 485 μM.min for IV AS). However, there were significant differences in both groups among the ePD parameters C_max_ and AUC_0–20_ where C _max_, of IV AS (20.7 μM) was 2.7 times greater than that of IV AL (7.76 μM) and AUC_0–20_ of IV AS (242 μM.min) was two-fold greater than IV AL (115 μM.min).

**Table 3:** Pharmacokinetic parameters based on combined parent drug and metabolite levels and ex vivo pharmacodynamics (ePD)

Pharmacokinetics		Rx	Ex vivo pharmacodynamic^a^				Combined P + M				Ex vivo potency		% AUC_0–20_	
Parameter	Unit		Median	25th IQT	75th IQT	p^b^	Median	25th IQT	75th IQT	p^b^	ePD/PK	AS/AL	AUC_0–∞_	
C_max_^c^	mM	IV AL	7.76	7.09	8.13	<0.001	64.6	62.7	68.5	<0.001	0.120			
		IV AS	20.7	15.3	30.5		35.2	31.3	44.4		0.587	4.89		
t_1/2_^d^	min	IV AL	29.6	26.3	38.0	0.7	36.3	30.6	44.8	0.02	0.815			
		IV AS	29.9	23.4	33.2		29.1	23.7	31.4		1.028	1.26		
AUC_0–∞_^e^	mM min	IV AL	426	365	521	0.8	4911	3997	5827	<0.001	0.088			
		IV AS	485	297	586		764	698	926		0.643	7.31		
AUC_0–20_^f^	mM min	IV AL	115	109	121	<0.001	1051	1007	1107	<0.001	0.110		21.4	
		IV AS	242	172	348		404	373	502		0.598	5.44	52.9	
Cl^g^	L kg^−1^ min^−1^	IV AL	48.5	39.5	57.0	0.83								
		IV AS	43.0	35.7	70.6									
V_z_^h^	L kg^−1^	IV AL	2.14	1.91	2.32	0.57								
		IV AS	1.84	1.41	2.87									

^a^Concentration reported as DHA equivalents

^b^Mann Whitney Test

^c^ *C*_*max*_ maximum concentration of drug reached in plasma

^d^ *t*_*1/2*_ elimination half-life

^e^ *AUC*_*0–∞*_ area under the concentration–time curve from zero to infinity

^f^ *AUC*_*0–20*_ area under the concentration–time curve from zero to 20 min

^g^ *Cl* apparent total body clearance

^h^ *V*_*z*_ volume of distribution

Figure 3 indicated that the anti-malarial activity of IV AS was contributed by both the parent drug AS and its active metabolite DHA until 20 min post-dose when the major compound in plasma transitioned to DHA. Parent drug AS was below the detection limit beyond 1 h post-dose and DHA was detected at its highest level as early as 5 min post-dose. As a result, AUC_0–20_ was 97 % of AUC_0–∞_ for AS and 53 % for combined AS + DHA (Tables 2, 3). On the other hand, although OHAL metabolite was detected by HPLC-ECD, only AL was responsible for plasma anti-malarial activity (Fig. 3) and the AUC_0–20_ compared to the corresponding AUC_0–∞_ for AL and AL + OHAL which were comparable at 24.6 and 21.4 % respectively (Tables 2, 3). Furthermore, no DHA was found in plasma from either healthy or infected monkeys treated with IV AL.

Based on testing against the W2 clone, the in vitro potency of AS was comparable to DHA and was 4–5 times greater than that of AL. Since AS and its metabolite DHA contributed to the plasma anti-malarial activity, ex vivo potency was then determined from the AUC_0–∞_ ratio between ePD and PK of the combined parent and its metabolite (Table 3). Thus, the ex vivo potency of IV AS (0.643) was sevenfold greater than IV AL (0.088).

### Pharmacokinetic/pharmacodynamic correlation analysis (least square regression)

#### PK/ePD

Of all the parameters estimated, there were very strong correlations between PK and ePD for both formulations (Table 4). Only the parent drug AL had a linear relationship between the ePD parameters and t_1/2_ and AUC_0–∞_ (r^2^ > 0.95, p < 0.0001). In contrast, t_1/2_ of DHA and AUC_0–∞_ of AS + DHA for IV AS correlated linearly with ePD and to a smaller degree (r^2^ > 0.74, p < 0.001 and r^2^ > 0.85, p < 0.0002, respectively). As expected, the AUC_0–20_ values of the parent components of both IV preparations correlated well with ePD parameters (AL: r^2^ 0.75, p 0.005 and AS: r^2^ 0.80, p 0.0004). Only the C_max_ of AS, but not AL, strongly correlated with ePD parameters (r^2^ 0.81, p 0.0004).

**Table 4:** Correlation between PK and ePD

PK-ePD linear regression		IV AL				IV AS			
Parameter	Analyte	a	b	r^2^	p	a	b	r^p^	p
C_max_ (mM)	Parent					1.04	7.45	0.807	<0.001
t_1/2_ (min)	Parent	0.69	7.89	0.964	<0.001				
	Metabolite					0.72	9.09	0.743	0.001
AUC_0–∞_ (mM min)	Parent	7.88	124	0.951	<0.001				
	Combined					1.12	265	0.847	<0.001
AUC_0–20_ (mM min)	Parent	8.09	32.7	0.751	0.005	0.94	76.3	0.803	<0.001

PK = a*ePD + b

#### PK or ePD)/iPD

**Fig. 4:**
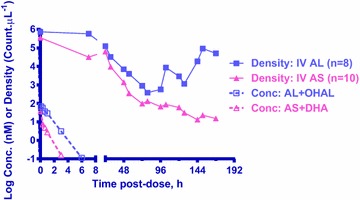
Comparison of mean parasite density and drug concentration–time profiles. Mean parasite density-time (iPD, *solid line*) and concentration–time (PK, *dotted line*) profiles of combined parent + metabolite for both parenteral regimens

**Fig. 5:**
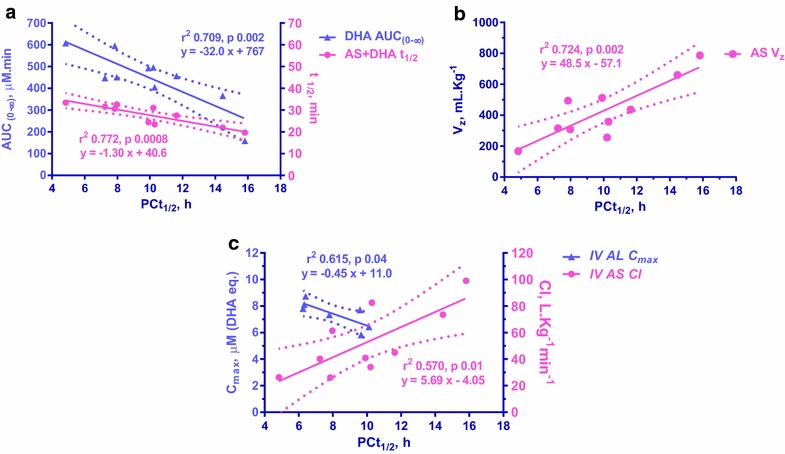
Pharmacokinetic-pharmacodynamic correlation. The best three PK-iPD correlations are shown in (**a**, **b**). All the PK parameters following IV AS treatment: t_1/2_ of AS + DHA (r^2^ 0.772), the V_z_ of AS (r^2^ 0.724), and AUC_0–∞_ of DHA (r^2^ 0.709) showed strong and significant (p ≤ 0.002) correlation with the parasite clearance half-life (PCt_1/2_). The ePD-iPD correlation following both treatments is shown in (**c**). The PCt_1/2_ significantly (p ≤ 0.04) and inversely correlated well with the C_max_ of the effect kinetics following IV AL (r^2^ 0.615) and correlated with the clearance (Cl) of the effect kinetics following IV AS (r^2^ 0.570). *Solid line* denotes linear regression and *dotted line* denotes 95 % confidence interval

The concentration–time profiles (PK) of parent + metabolite and the parasite density-time profile (iPD) for both parenteral regimens were shown in Fig. 4. The PK and ePD parameters for IV AS significantly correlated with those of iPD; while only the ePD of IV AL showed significant correlation with iPD (Table 5). For IV AS, all PK parameters estimated, C_max_, AUC_0–∞_, t_1/2_, Cl and V_z_, were significantly correlated well with the estimated parasite clearance parameters—PCt_1/2_, PCR, and PCT. The PK of parent AS showed its V_z_, C_max_ and Cl correlated well with PCt_1/2_ (r^2^ 0.72, p 0.002), PCR (r^2^ 0.60, p 0.009) and PCT (r^2^ 0.53, p 0.02), respectively. The PK of DHA showed that AUC_0–∞_ inversely and strongly correlated with PCt_1/2_ (r^2^ 0.71, p 0.002) and PCT (r^2^ 0.61, p 0.008) and t_1/2_ correlated with PCR (r^2^ 0.58, p 0.01). The PK of combined AS + DHA showed its t_1/2_ inversely and strongly correlated with PCt_1/2_ (r^2^ 0.77, p < 0.001), C_max_ correlated with PCR (r^2^ 0.62, p 0.007), and AUC_0–∞_ inversely correlated with PCT (r^2^ 0.60, p 0.009). Lastly, the effect kinetic (ePD) of IV AS revealed that Cl and AUC_0–∞_ correlated with PCt_1/2_ (r^2^ 0.57, p 0.01) and PCR (r^2^ 0.52, p 0.02), respectively. On the other hand for IV AL, only C_max_ correlated with PCt_1/2_ and PCR (r^2^ 0.61, p 0.04). Correlation analysis for PCT was not possible due to limited data points (n = 2). Figure 5 show the best PK-iPD with strong correlation (r^2^ > 0.7) for IV AS and ePD-iPD for both regimens.

**Table 5:** Linear regression analyses between PK or *ePD* and iPD

Treatment	Compound	PK (*ePD*)	Unit	iPD	Unit	r^2^	p	a	b
IV AS	AS	V_z_	mL kg^−1^	PCt_1/2_	h	0.724	0.002	48.5	−57.2
		C_max_	mM	PCR	min	0.596	0.009	4.83	8.45
		Cl	L kg^−1^ min^−1^	PCT	Day	0.534	0.02	12.5	−12.2
	DHA	AUC_0–∞_	mM min	PCt_1/2_	h	0.709	0.002	−32.0	767
		AUC_0–∞_	mM min	PCT	Day	0.608	0.008	−48.9	780
		t_1/2_	min	PCR	min	0.576	0.01	2.30	19.3
	AS + DHA	t_1/2_	min	PCt_1/2_	h	0.772	0.0008	−1.30	40.6
		C_max_	mM	PCR	min	0.619	0.007	5.09	14.0
		AUC_0–∞_	mM min	PCT	Day	0.596	0.009	−98.5	1487
	BA	*Cl*	L kg^−1^ min^−1^	PCt_1/2_	h	0.571	0.01	5.69	−4.08
		*AUC*_*0–∞*_	mM min	PCR	min	0.516	0.02	87.6	71.1
IV AL	BA	*C*_*max*_	mM	PCt_1/2_	h	0.615	0.04	0.447	11.0
		*C*_*max*_	mM	PCR	min	0.611	0.04	0.665	3.82

$$ {\text{PK}} = {\text{a}} * {\text{iPD}} + {\text{b}}, $$ or $$ {\text{ePD}} = {\text{a}} * {\text{iPD}} + {\text{b}} $$

## Discussion

While direct comparison of safety and efficacy were complicated by experimental factors, IV AS proved to have superior potency and more rapid parasite clearance compared to IV AL. Similar to prior studies in humans [15, 18, 19], PK/PD relationships for AS in monkeys revealed that the parent drug AS was rapidly converted to DHA and undetectable as early as 1 h post-dose. Activity and metabolic conversion were rapid. While AS was at fourfold higher concentration at 5 min post dose, only DHA remained responsible for the observed anti-malarial activity roughly 20 min post-dose when plasma concentrations of DHA far exceeded AS. The t_1/2_ of DHA was at least ninefold longer than AS. This is consistent with prior observations in healthy human studies attributing the effectiveness of AS in part to its rapid and extensive hydrolysis to DHA, achieving high initial drug levels [18].

Challenges inherent to the model limit experimental interpretation to some degree. The two experimental groups were inoculated and dosed separately rather than concurrently to allow sufficient time to adequately perform all procedures. Based on previous model development, the number of *P. coatneyi* parasites was expected to increase rapidly on study day 6. Therefore, blood parasitaemia was not measured prior to treatment in order to limit overall blood draws. Animals in the IV AS group were dosed first and morning parasitaemia levels varied between 0.5 and 10.1 %, while parasitaemia in IV AL animals was between 7.1 and 20.2 %, with roughly twofold higher median parasitaemia in the IV AL group (12 %) compared to IV AS (6.6 %) at initial dose. To some extent, this makes a meaningful in vivo PK/PD comparison difficult. Moreover, there were two deaths in the IV AL group limiting available data from those animals. Baseline parasitaemias in these animals (13.2 and 13.0 %) were comparable to those of other animals. Both monkeys died when their parasite density reached 1.2 × 10^6^ parasites μL^−1^. Parasitaemias were 15 and 20 % respectively at 1 h apart suggesting that malaria was at the very least a major contributor to their demise. Although the aetiology of death could not be determined definitively, pathologic examination favored a systemic inflammatory syndrome consistent with severe malaria over drug toxicity.

The *P. coatneyi* model requires splenectomy in order to ensure adequate parasite infection and determine efficacy, and the results should be interpreted in light of this consideration. Normally, the spleen removes abnormal erythrocytes and intra-erythrocytic inclusions, including dead parasites which are removed from erythrocytes without erythrocyte destruction by the spleen [20]. Infected RBCs may persist longer and likely delayed parasite clearance compared to what would have been observed in spleen-intact animals. In a tissue distribution study in rats, 1 h following IV administration of AS, high levels of AS were found in brain, fat, intestine and serum, with comparatively low levels in other tissues including liver, kidney, testicle, muscle, fat, heart, eye, spleen and lung [21]. Splenectomy was unlikely to have had a significant effect on the PK parameters and metabolism of AS or AL given the short half-life of both drugs.

In light of these findings, the human-rhesus dose conversion factor can be derived based on PK parameters.

Let AUC in human = H_A_, AUC in Rhesus = R_A_, dose in human = H_D_, dose in Rhesus = R_D_

$$ {\text{AUC per unit dose in human}} = {\text{H}}_{\text{A}} /{\text{H}}_{\text{D}} $$

$$ {\text{AUC per unit dose in Rhesus}} = {\text{R}}_{\text{A}} /{\text{R}}_{\text{D}} $$

$$ {\text{Human}}/{\text{Rhesus conversion factor based on AUC per unit dose}} = {\text{F}} $$

$$ {\text{F}} = {\text{AUC per unit dose in human}}/{\text{AUC per unit dose in Rhesus}} $$

$$ = {\text{H}}_{\text{A}} /{\text{H}}_{\text{D}} * {\text{R}}_{\text{D}} /{\text{R}}_{\text{A}} $$

1 $$ {\text{F}} * {\text{H}}_{\text{D}} * {\text{R}}_{\text{A}} = {\text{H}}_{\text{A}} * {\text{R}}_{\text{D}} $$

Since equal AUC for human and Rhesus is desired,

$$ {\text{H}}_{\text{A}} = {\text{R}}_{\text{A}} $$

Then,

2 $$ {\text{F}} = {\text{R}}_{\text{D}} /{\text{H}}_{\text{D}} $$

The ePD of IV AS in uncomplicated *P. coatneyi*-infected rhesus monkey (8 mg kg^−1^ dose) can be directly compared to that of patients with uncomplicated falciparum malaria (2 mg kg^−1^ dose) [22]. Using total drug exposure, AUC values in human vs rhesus (636 vs 476 µM min), the AUC per unit dose for human was 318 and that of rhesus was 59.5. Therefore, the approximate inter-species conversion factor for human in relation to rhesus is 5.34.

From Eq. (2), if the human dose = 2 mg kg^−1^,

Rhesus equivalent dose = 5.34*2

=10.7 mg kg^−1^

Vice versa, if the Rhesus dose = 8 mg kg^−1^,

Human equivalent dose = 8/5.34

=1.50 mg kg^−1^.

**Table:** 

Subject	Reference	Cmax, µmol L^−1^	AUC, µmol min L^−1^	t_1/2_, min	Cl, L h^−1^ kg^−1^	Vd, L kg^−1^
Human	Newton, 2000	29.0 (7.69–249)	636 (524–748)	43.8 (37.2–49.8)	0.83 (0.70–0.96)	0.27 (0.20–0.34)
Rhesus	Current study	22.4 (16.7–28.2)	476 (346–607)	29.2 (25.3–33.1)	3.18 (2.25–4.11)	2.13 (1.59–2.66)

**Table:** 

Subject	AUC, µmol min L^−1^	AUC/dose		Dose, mg kg^−1^
Human	636	318	Rhesus equi.	10.7
Rhesus	476	59.5	Human equi.	1.50
H/R		5.34		

Values are mean (95 % Confidence interval)

Studies in multiple species [23, 24] have demonstrated limited conversion of AL to DHA. However, we found no DHA in rhesus plasma at all, instead detecting the OHAL metabolite by HPLC-ECD in accordance with a previous report suggesting that this is a product of rhesus monkey microsomes [25]. This finding indicates that the parent drug AL alone contributed to blood stage anti-malarial activity, and is concordant with earlier work also demonstrating in vitro conversion of AL into OHAL in human liver microsomes [25, 26]. Furthermore, the effect of IV AS and IV AL on *P. coatneyi* recrudescence seen here in non-human primates was similar to that seen for *Plasmodium vinckei* in rodents [27], suggesting the possibility that both AS and AL may trigger in vivo parasites dormancy in NHPs. This has been postulated as an underlying mechanism of treatment failure for artemisinin monotherapy [28]. Nevertheless, recrudescence due to short-course artemisinin treatment can be overcome by the use of combination therapy with slow acting anti-malarial agents [29].

Correlation between drug exposure (PK) and drug effect (PD) for artemisinin compounds has been challenging due to rapid clearance of drug, unclear mechanism of action, and sequestration in red blood cells combined with the inability to measure intraerythrocytic drug levels. Parasite clearance, a function of host-parasite-drug interactions, is a good measure of the in vivo drug effect. Davis et al. [30] reported only a borderline significant inverse correlation between AUC and PC50 following IV AS therapy for severe malaria in a 2001 study. Ten years later, White [31] demonstrated that the parasite clearance time was associated with parasite density, potentially confounding PK/PD correlations with artemisinins, and this was borne out in subsequent field studies of oral artesunate monotherapy [32]. Bakshi et al. [33] developed a PK/PD in vitro system and found that the efficacy of artemisinin depended on C_max_ while that of chloroquine depended on its time above minimum inhibitory concentration, T_MIC_, which were confirmed in an in vivo *Plasmodium berghei*-mouse model. Pooled analysis of data from severe malaria patients following IV AS revealed there was no significant association between PCt_1/2_ and post hoc population PK estimates. Lastly, children have been found to have lower DHA exposure than adults [34], with a recent study by Hawkes et al. [35] concluding that slower parasite clearance in children relative to adults may be explained by lower DHA exposure. However, they did not observe any correlation between DHA exposure and parasite clearance.

In present study using uncomplicated *P. coatneyi*-Rhesus monkey malaria model, PK-PD analysis of IV AS reveals an inverse linear correlation of DHA’s AUC with both PCt_1/2_ and PCT (Table 5) with longer PCt_1/2_ and PCT resulting from lower DHA exposure. This is consistent with Hawkes’ conclusion in humans suggesting that the rhesus model mimics human PK/PD for IV AS. Moreover, although AS is rapidly metabolized to DHA following IV administration, parasite clearance appears to be contributed by both components (Fig. 5) and the efficacy of IV AS depends on the t_1/2_ of combined AS + DHA > V_z_ of AS > AUC of DHA. In addition, IV AS had strong correlation between PK and iPD (in vivo drug effect against *P. coatneyi*-infected monkey) but not ePD (ex vivo drug effect against *P. falciparum* blood stage) and iPD suggesting that AS and DHA both contributed to in vivo activity against both blood and tissue parasite stages. On the other hand effect kinetic parameter C_max_ for IV AL was inversely correlated with PCt_1/2_. The strong correlation between PK and ePD as well as ePD and iPD implied that the in vivo activity of AL may be predominantly blood stage activity.

## Conclusion

In a non-human primate model of uncomplicated malaria, IV AS had greater ex vivo potency against *P. falciparum* with higher anti-*P. falciparum* activity during the first 20 min of drug administration. The model demonstrated that parasite clearance was dependent on drug exposure including both AUC and C_max_ for both artemisinin regimens. While the efficacy of IV AS depended on the t_1/2_ of combined AS + DHA, the V_z_ of AS, and the AUC of DHA, efficacy of IV AL depended largely on its effect kinetic parameter C_max_, and was largely restricted to parent drug activity. As a result, IV AS was selected for further development for the treatment of severe malaria under an Investigational New Drug application (IND) held by the U.S. Army Office of the Surgeon General. In the United States, it is currently available to patients under an IND treatment protocol maintained at the Centers for Disease Control [36] and by the military.

## Additional files

10.1186/s12936-016-1456-6 The concentration–time profiles as measured by HPLC-ECD following intravenous artelinate (lysine salt) 11.8 mg/kg in healthy (n = 10) and *P. coatneyi* infected rhesus monkeys (n = 8). Values are mean and 95 % confidence interval of concentration in μmole L^−1^.
10.1186/s12936-016-1456-6 The concentration–time profiles as measured by HPLC-ECD following intravenous artesunate (sodium salt) 8.0 mg/kg in healthy and *P. coatneyi* infected rhesus monkeys (n = 10 in each group). Values are mean and 95 % confidence interval of concentration in μmole L^−1^.
10.1186/s12936-016-1456-6 The antimalarial effect-time profiles as measured by bioassay following intravenous artelinate (lysine salt) 11.8 mg/kg and artesunate (sodium salt) 8.0 mg/kg in healthy and *P. coatneyi* infected rhesus monkeys. Values are mean and 95 % confidence interval of antimalarial activity expressed as DHA equi. in μmole L^−1^.
10.1186/s12936-016-1456-6 Parasite-time profiles and recrudescence (R) day following once daily dose of IV AL 11.8, 5.6, 5.6 mg kg^−1^ and IV AS 8.0, 4, 4 mg kg^−1^ for 3 days, showing mean and 95 % confidence in plasma of *P. coatneyi* infected rhesus monkeys including quinine control for each treatment and percentage (mean and 95 %CI) of parasite reduction after treatment.
10.1186/s12936-016-1456-6 Pharmacokinetic parameters of parent drug (AL) and metabolite (2-OHAL) and effect kinetic parameters following I.V. AL/Lys 11.8 mg/kg, in healthy and *P. coatneyi* infected rhesus monkeys with their means, 95 % confidence intervals, and coefficient of variation (%CV) values.
10.1186/s12936-016-1456-6 Pharmacokinetic parameters of parent drug (AS) and metabolite (DHA) and effect kinetic parameters following I.V. AS/Na 8 mg/kg, in healthy and *P. coatneyi* infected rhesus monkeys with their means, 95 % confidence intervals, and coefficient of variation (%CV) values.
10.1186/s12936-016-1456-6 Direct comparison of the PK/PD profiles of I.V. AL/lys between healthy self-controls (n = 10) and infected (n = 8) rhesus monkeys. a) Parent drug AL and b) its metabolite 2-OHAL profiles as measured by HPLC-ECD, and c) bioactivity profile as measured by bioassay. Values are mean + 95 % CI.
10.1186/s12936-016-1456-6 Direct comparison of the PK/PD profiles of I.V. AS/Na between healthy self-controls (n = 10) and infected (n = 10) rhesus monkeys. a) Parent drug AS and b) its metabolite DHA profiles as measured by HPLC-ECD, and c) bioactivity profile as measured by bioassay. Values are mean + 95 % CI.
