# Supplementary material for: Comparative pharmacokinetics and pharmacodynamics of intravenous artelinate versus artesunate in uncomplicated Plasmodium coatneyi-infected rhesus monkey model
Source: Malar J. 2016 Sep 6;15(1):453. doi: 10.1186/s12936-016-1456-6 (PMC5011932; doi:10.1186/s12936-016-1456-6)
Supplement: Supplementary file 1 — 10.1186/s12936-016-1456-6 The concentration–time profiles as measured by HPLC-ECD following intravenous artelinate (lysine salt) 11.8 mg/kg in healthy (n = 10) and P. coatneyi infected rhesus monkeys (n = 8). Values are mean and 95 % confidence interval of concentration in μmole L−1. [file 12936_2016_1456_MOESM1_ESM.docx]

**Additional file 1**. The concentration-time profiles as measured by HPLC-ECD following intravenous artelinate (lysine salt) 11.8 mg/kg in healthy (n = 10) and *P. coatneyi* infected rhesus monkeys (n = 8). Values are mean and 95% confidence interval of concentration in μmole L^-1^

|  | **AL** (Parent drug) | | | | **2-OHAL** (Metabolite) | | | |
| --- | --- | --- | --- | --- | --- | --- | --- | --- |
| Time | Healthy | | Infected | | Healthy | | Infected | |
| **h** | **Mean** | 95% CI | **Mean** | 95% CI | **Mean** | 95% CI | **Mean** | 95% CI |
| **0** | **0** |  | **0** |  | **0** |  | **0** |  |
| **0.08** | **61.58** | 56.63-66.53 | **62.96** | 56.64-69.28 | **3.33** | 2.09-4.57 | **4.64** | 2.06-7.23 |
| **0.33** | **43.27** | 39.24-47.31 | **46.68** | 40.60-52.76 | **9.12** | 6.91-11.33 | **10.03** | 7.70-12.37 |
| **0.67** | **26.12** | 22.45-29.79 | **26.79** | 21.38-32.19 | **8.97** | 7.40-10.53 | **9.39** | 5.93-12.86 |
| **1.0** | **16.73** | 13.38-20.09 | **21.04** | 16.57-25.51 | **7.03** | 5.78-8.28 | **8.21** | 5.13-11.30 |
| **3.0** | **0.68** | 0.23-1.14 | **1.24** | 0.48-2.01 | **0.49** | 0.14-0.83 | **1.87** | 0.66-3.07 |
| **6.0** | **0** | 0 | **0.05** | -0.05-0.15 | **0** | 0 | **0.06** | -0.01-0.13 |
