# Supplementary material for: Comparative pharmacokinetics and pharmacodynamics of intravenous artelinate versus artesunate in uncomplicated Plasmodium coatneyi-infected rhesus monkey model
Source: Malar J. 2016 Sep 6;15(1):453. doi: 10.1186/s12936-016-1456-6 (PMC5011932; doi:10.1186/s12936-016-1456-6)
Supplement: Supplementary file 2 — 10.1186/s12936-016-1456-6 The concentration–time profiles as measured by HPLC-ECD following intravenous artesunate (sodium salt) 8.0 mg/kg in healthy and P. coatneyi infected rhesus monkeys (n = 10 in each group). Values are mean and 95 % confidence interval of concentration in μmole L−1. [file 12936_2016_1456_MOESM2_ESM.docx]

**Additional file 2**. The concentration-time profiles as measured by HPLC-ECD following intravenous artesunate (sodium salt) 8.0 mg/kg in healthy and *P. coatneyi* infected rhesus monkeys (n = 10 in each group). Values are mean and 95% confidence interval of concentration in μmole L^-1^

|  | **AS** (Parent drug) | | | | **DHA** (Metabolite) | | | |
| --- | --- | --- | --- | --- | --- | --- | --- | --- |
| Time | Healthy | | Infected | | Healthy | | Infected | |
| **H** | **Mean** | 95% CI | **Mean** | 95% CI | **Mean** | 95% CI | **Mean** | 95% CI |
| **0** | **0** |  | **0** |  | **0** |  | **0** |  |
| **0.08** | **26.56** | 20.97-32.14 | **30.79** | 24.11-37.48 | **7.89** | 6.38-9.41 | **6.74** | 6.24-7.25 |
| **0.33** | **2.89** | -0.17-5.95 | **1.34** | 0.88-1.81 | **6.75** | 5.99-7.51 | **5.92** | 5.25-6.59 |
| **0.67** | **0.11** | 0.02-0.21 | **0.64** | -0.43-1.71 | **4.51** | 3.79-5.24 | **4.21** | 3.46-4.96 |
| **1.0** | - | - | **0.05** | -0.04-0.14 | **3.52** | 2.83-4.20 | **2.68** | 2.11-3.25 |
| **3.0** | - | - | 0 | 0 | **0.31** | 0.18-0.44 | **0.16** | 0.09-0.23 |
| **6.0** | - | - | 0 | 0 | **0** | 0-0.01 | **0** | 0 |
