# Supplementary material for: Comparative pharmacokinetics and pharmacodynamics of intravenous artelinate versus artesunate in uncomplicated Plasmodium coatneyi-infected rhesus monkey model
Source: Malar J. 2016 Sep 6;15(1):453. doi: 10.1186/s12936-016-1456-6 (PMC5011932; doi:10.1186/s12936-016-1456-6)
Supplement: Supplementary file 3 — 10.1186/s12936-016-1456-6 The antimalarial effect-time profiles as measured by bioassay following intravenous artelinate (lysine salt) 11.8 mg/kg and artesunate (sodium salt) 8.0 mg/kg in healthy and P. coatneyi infected rhesus monkeys. Values are mean and 95 % confidence interval of antimalarial activity expressed as DHA equi. in μmole L−1. [file 12936_2016_1456_MOESM3_ESM.docx]

**Additional file 3**. The antimalarial effect-time profiles as measured by bioassay following intravenous artelinate (lysine salt) 11.8 mg/kg and artesunate (sodium salt) 8.0 mg/kg in healthy and *P. coatneyi* infected rhesus monkeys. Values are mean and 95% confidence interval of antimalarial activity expressed as DHA equi. in μmole L^-1^.

|  | I.V. **Artelinate** /Lys | | | | I.V **Artesunate**/Na | | | |
| --- | --- | --- | --- | --- | --- | --- | --- | --- |
| Time | Healthy (n=10) | | Infected (n=8) | | Healthy (n=10) | | Infected (n=10) | |
| **h** | **Mean** | 95% CI | **Mean** | **95% CI**  **6.98-8.18**  **4.61-6.04**  **2.98-4.18**  **2.01-3.14**  **0.07-0.32**  **0**  **Mean** | **Mean** | 95% CI | **Mean** | 95% CI |
| **0** | **0** |  | **0** |  | **0** |  | **0** |  |
| **0.08** | **5.69** | 4.66-6.72 | **7.58** | 6.98-8.18 | **17.94** | 14.6-21.3 | **22.45** | 16. 7-28.2 |
| **0.33** | **4.09** | 3.57-4.59 | **5.32** | 4.61-6.04 | **5.77** | 2.79-8.74 | **4.28** | 3.09-5.47 |
| **0.67** | **2.56** | 2.06-3.06 | **3.58** | 2.98-4.18 | **3.16** | 2.53-3.78 | **3.19** | 1.88-4.50 |
| **1.0** | **1.75** | 1.31-2.19 | **2.58** | 2.01-3.14 | **1.75** | 1.07-2.43 | **1.58** | 1.15-2.00 |
| **3.0** | **0.07** | 0.03-0.12 | **0.19** | 0.07-0.32 | **0.22** | 0.10-0.33 | **0.12** | 0.06-0.17 |
| **6.0** | **0** | 0 | **0** | 0 | **0** | 0-0.01 | **0** | 0 |
