# Supplementary material for: Comparative pharmacokinetics and pharmacodynamics of intravenous artelinate versus artesunate in uncomplicated Plasmodium coatneyi-infected rhesus monkey model
Source: Malar J. 2016 Sep 6;15(1):453. doi: 10.1186/s12936-016-1456-6 (PMC5011932; doi:10.1186/s12936-016-1456-6)
Supplement: Supplementary file 4 — 10.1186/s12936-016-1456-6 Parasite-time profiles and recrudescence (R) day following once daily dose of IV AL 11.8, 5.6, 5.6 mg kg−1 and IV AS 8.0, 4, 4 mg kg−1 for 3 days, showing mean and 95 % confidence in plasma of P. coatneyi infected rhesus monkeys including quinine control for each treatment and percentage (mean and 95 %CI) of parasite reduction after treatment. [file 12936_2016_1456_MOESM4_ESM.docx]

**Additional file 4**. Parasite-time profiles and recrudescence (R) day following once daily dose of IV AL 11.8, 5.6, 5.6 mg.kg^-1^ and IV AS 8.0, 4, 4 mg.kg^-1^ for 3 days, showing mean and 95% confidence in plasma of *P. coatneyi* infected rhesus monkeys including quinine control for each treatment and percentage (mean and 95%CI) of parasite reduction after treatment.

|  | **IV AL** | | | | | **IV AS** | | | | |
| --- | --- | --- | --- | --- | --- | --- | --- | --- | --- | --- |
| Time post-dose, Day | Parasite/μL | | Parasite Reduction, % | | | Parasite/μL | | Parasite Reduction, % | | |
|  | Mean | 95%CI | Mean | 95%CI | IM QN | Mean | 95%CI | Mean | 95%CI | IM QN |
| 0.00 | 773,172 | 91,451 | 0.00 |  | 0.0 | 353,193 | 142,236 | 0.00 |  | 0.0 |
| 0.29 | 704,770 | 82,906 | 20.4 | 11.0 | 9.91 | 32,080 | 16,007 | 88.6 | 4.8 | 68.1 |
| 1.00 | 124,294 | 58,944 | 82.2 | 9.65 | 38.4 | 63,163 | 41,263 | 81.1 | 8.7 | 77.5 |
| 1.29 | 32,703 | 13,439 | 95.2 | 2.49 | 82.7 | 9,364 | 7,846 | 97.1 | 1.4 | 97.7 |
| 2.00 | 6,978 | 6,207 | 98.9 | 1.09 | 97.3 | 1,383 | 946 | 99.4 | 0.3 | 99.8 |
| 2.29 | 3,976 | 3,443 | 99.4 | 0.60 | 97.5 | 345 | 236 | 99.8 | 0.1 | 99.9 |
| 3.00 | 895 | 773 | 99.9 | 0.14 | 98.0 | 96 | 37 | 100.0 | 0.0 | 100 |
| 3.29 | 377 | 387 | 99.9 | 0.07 | 99.4 | 132 | 54 | 99.9 | 0.0 | 100 |
| 4.00 | 568 | 861 | 99.9 | 0.15 | 99.7 | 66 | 43 | 100.0 | 0.0 | 100 |
| 4.29 | 8,704 | 16,732 | 98.5 | 2.87 | 99.9 | 87 | 51 | 100.0 | 0.0 | 100 |
| 5.00 | 2,880 | 4,815 | 99.5 | 0.83 | 99.9 | 60 | 54 | 100.0 | 0.0 | 100 |
| 5.29 | 1,141 | 1,614 | 99.8 | 0.28 | 100 | 31 | 29 | 100.0 | 0.0 | 100 |
| 6.00 | 18,619 | 35,369 | 96.8 | 6.06 | 100 | 13 | 15 | 100.0 | 0.0 | 100 |
| 6.29 | 92,458 | 150,342 | 84.4 | 25.8 | 100 | 23 | 23 | 100.0 | 0.0 | 100 |
| 7.00 | 51,036 | 78,844 | 91.4 | 13.5 | 100 | 15 | 17 | 100.0 | 0.0 | 100 |
| 8.00 |  |  |  |  |  | 4 | 6 | 100.0 | 0.0 | 100 |
| 9.00 |  |  |  |  |  | 2 | 5 | 100.0 | 0.0 | 100 |
| 10.00 |  |  |  |  |  | 0 |  | 100.0 |  | 100 |
| R, Day | 6.50 | 0.44 |  |  |  | 13 | 1.73 |  |  |  |
| n | 2 | |  |  |  | 10 | |  |  |  |
