# Supplementary material for: Comparative pharmacokinetics and pharmacodynamics of intravenous artelinate versus artesunate in uncomplicated Plasmodium coatneyi-infected rhesus monkey model
Source: Malar J. 2016 Sep 6;15(1):453. doi: 10.1186/s12936-016-1456-6 (PMC5011932; doi:10.1186/s12936-016-1456-6)
Supplement: Supplementary file 5 — 10.1186/s12936-016-1456-6 Pharmacokinetic parameters of parent drug (AL) and metabolite (2-OHAL) and effect kinetic parameters following I.V. AL/Lys 11.8 mg/kg, in healthy and P. coatneyi infected rhesus monkeys with their means, 95 % confidence intervals, and coefficient of variation (%CV) values. [file 12936_2016_1456_MOESM5_ESM.docx]

**Additional file 5**. Pharmacokinetic parameters of parent drug (AL) and metabolite (2-OHAL) and effect kinetic parameters following I.V. AL/Lys 11.8 mg/kg, in healthy and *P. coatneyi* infected rhesus monkeys with their means, 95% confidence intervals, and coefficient of variation (%CV) values.

| Parameter estimates | **Healthy** rhesus (n = 10) | | | **Infected** rhesus (n = 8) | | |
| --- | --- | --- | --- | --- | --- | --- |
| **AL** (parent) | **Mean** | 95%CI | %CV | **Mean** | 95%CI | %CV |
| **C_max_** (μmole L^-1^) | **61.6** | 56.6-66.5 | 13.0 | **61.5** | 55.1-67.9 | 15.0 |
| **AUC_(0_-_inf)_** (μmole min L^-1^) | **3,039** | 2,498-3,579 | 28.7 | **3,392** | 2,562-4,221 | 35.2 |
| **t _1/2, z_** (min) | **25.3** | 21.7-29.0 | 23.0 | **29.6** | 25.0-34.1 | 22.1 |
| **V_z_** (L kg ^-1^) | **0.26** | 0.22-0.30 | 24.1 | **0.28** | 0.23-0.33 | 27.1 |
| **Cl** (L hr^-1^ kg ^-1^) | **0.45** | 0.34-0.56 | 39.7 | **0.43** | 0.28-0.57 | 49.4 |
| **AUC _0-20_** (μmole min L^-1^) | **940** | 866-1,014 | 12.7 | **954** | 851-1,056 | 15.5 |
| **%AUC_20_** | **33.4** | 27.1-39.7 | 30.6 | **31.5** | 23.3-39.6 | 37.3 |
| **2-OHAL (**metabolite**)** |  |  |  |  |  |  |
| **C_max_** (μmole L^-1^) | **9.57** | 7.43-11.71 | 36.0 | **11.7** | 9.08-14.3 | 32.1 |
| **AUC_(0_-_inf)_** (μmole min L^-1^) | **843** | 686-1,001 | 30.2 | **1,363** | 854-1,872 | 53.9 |
| **t _1/2, z_** (min) | **33.8** | 25.1-42.4 | 41.3 | **44.0** | 33.3-54.7 | 35.0 |
| **t _max_** (min) | **34.0** | 25.6-42.4 | 39.7 | **35.0** | 22.7-47.3 | 50.7 |
| **AUC _0-20_** (μmole min L^-1^) | **102** | 74-130 | 44.2 | **130** | 88-172 | 46.5 |
| **%AUC_20_** | **12.7** | 9.0-16.5 | 47.6 | **11.0** | 6.4-15.6 | 60.0 |
| **Bioactivity** (DHA equi.) |  |  |  |  |  |  |
| **C_max_** (μmole L^-1^) | **5.69** | 4.66-6.72 | 29.2 | **7.51** | 6.85-8.18 | 12.8 |
| **AUC_(0_-_inf)_** (μmole min L^-1^) | **299** | 232-366 | 36.0 | **431** | 349-513 | 27.6 |
| **t _1/2, z_** (min) | **26.7** | 23.5-29.8 | 19.3 | **31.3** | 24.9-37.7 | 29.5 |
| **V_z_** (L kg ^-1^) | **2.90** | 2.31-3.50 | 33.0 | **2.17** | 1.96-2.37 | 13.5 |
| **Cl** (L hr^-1^ kg ^-1^) | **4.75** | 3.46-6.04 | 43.8 | **3.08** | 2.42-3.76 | 31.2 |
| **AUC _0-20_** (μmole min L^-1^) | **88** | 74-101 | 25.6 | **114** | 103-125 | 14.0 |
| **%AUC_20_** | **31.7** | 25.6-37.7 | 30.7 | **27.7** | 23.7-31.6 | 20.7 |
