# Supplementary material for: Comparative pharmacokinetics and pharmacodynamics of intravenous artelinate versus artesunate in uncomplicated Plasmodium coatneyi-infected rhesus monkey model
Source: Malar J. 2016 Sep 6;15(1):453. doi: 10.1186/s12936-016-1456-6 (PMC5011932; doi:10.1186/s12936-016-1456-6)
Supplement: Supplementary file 6 — 10.1186/s12936-016-1456-6 Pharmacokinetic parameters of parent drug (AS) and metabolite (DHA) and effect kinetic parameters following I.V. AS/Na 8 mg/kg, in healthy and P. coatneyi infected rhesus monkeys with their means, 95 % confidence intervals, and coefficient of variation (%CV) values. [file 12936_2016_1456_MOESM6_ESM.docx]

**Additional file 6**. Pharmacokinetic parameters of parent drug (AS) and metabolite (DHA) and effect kinetic parameters following I.V. AS/Na 8 mg/kg, in healthy and *P. coatneyi* infected rhesus monkeys with their means, 95% confidence intervals, and coefficient of variation (%CV) values.

| Parameter estimates | **Healthy** rhesus (n = 10) | | | **Infected** rhesus (n = 10) | | |
| --- | --- | --- | --- | --- | --- | --- |
| **AS** (Parent drug) | **Mean** | 95%CI | %CV | **Mean** | 95%CI | %CV |
| **C_max_** (μmole L^-1^) | **26.6** | 21.0-32.1 | 33.9 | **30.8** | 24.1-37.5 | 35.0 |
| **AUC_(0-inf)_** (μmole min L^-1^) | **312** | 237-388 | 38.9 | **336** | 251-421 | 41.0 |
| **t_1/2, z_** (min) | **4.17** | 3.46-4.88 | 27.5 | **4.43** | 3.03-5.83 | 51.02 |
| **V_z_** (L kg ^-1^) | **0.47** | 0.31-0.63 | 54.6 | **0.43** | 0.31-0.55 | 44.2 |
| **Cl** (L hr^-1^ kg ^-1^) | **4.70** | 3.33-6.08 | 47.3 | **4.38** | 3.10-5.66 | 47.2 |
| **AUC _0-20_** (μmole min L^-1^) | **287** | 228-347 | 33.3 | **318** | 249-387 | 35.1 |
| **%AUC_20_** | **93.9** | 87.9-99.9 | 10.3 | **96.6** | 91.8-101.3 | 7.9 |
| **DHA** (metabolite) |  |  |  |  |  |  |
| **C_max_** (μmole L^-1^) | **8.10** | 6.77-9.42 | 26.4 | **6.85** | 6.33-7.37 | 12.2 |
| **AUC_(0_-_inf)_** (μmole min L^-1^) | **557** | 469-645 | 25.4 | **447** | 368-525 | 28.3 |
| **t_1/2, z_** (min) | **35.9** | 31.2-40.6 | 21.2 | **30.0** | 26.7-33.2 | 17.5 |
| **AUC _0-20_** (μmole min L^-1^) | **130** | 110-149 | 24.3 | **112** | 103-121 | 12.9 |
| **%AUC_20_** | **23.8** | 21.0-26.5 | 18.5 | **27.0** | 21.9-32.2 | 30.6 |
| **Bioactivity** (DHA equi.) |  |  |  |  |  |  |
| **C_max_** (μmole L^-1^) | **18.3** | 15.1-21.5 | 28.5 | **22.5** | 16.7-28.2 | 41.5 |
| **AUC_(0_-_inf)_** (μmole min L^-1^) | **482** | 375-589 | 35.8 | **476** | 346-607 | 44.1 |
| **t_1/2, z_** (min) | **36.6** | 30.2-43.0 | 28.3 | **29.2** | 25.3-33.1 | 21.6 |
| **V_z_** (L kg ^-1^) | **2.70** | 1.72-3.67 | 58.4 | **2.13** | 1.59-2.66 | 40.5 |
| **Cl** (L hr^-1^ kg ^-1^) | **3.07** | 2.07-4.07 | 52.5 | **3.18** | 2.25-4.11 | 47.3 |
| **AUC _0-20_** (μmole min L^-1^) | **223** | 181-264 | 30.1 | **257** | 191-322 | 41.4 |
| **%AUC_20_** | **48.3** | 42.2-54.3 | 20.2 | **55.0** | 50.6-59.4 | 12.8 |
