# Supplementary material for: Comparative pharmacokinetics and pharmacodynamics of intravenous artelinate versus artesunate in uncomplicated Plasmodium coatneyi-infected rhesus monkey model
Source: Malar J. 2016 Sep 6;15(1):453. doi: 10.1186/s12936-016-1456-6 (PMC5011932; doi:10.1186/s12936-016-1456-6)
Supplement: Supplementary file 7 — 10.1186/s12936-016-1456-6 Direct comparison of the PK/PD profiles of I.V. AL/lys between healthy self-controls (n = 10) and infected (n = 8) rhesus monkeys. a) Parent drug AL and b) its metabolite 2-OHAL profiles as measured by HPLC-ECD, and c) bioactivity profile as measured by bioassay. Values are mean + 95 % CI. [file 12936_2016_1456_MOESM7_ESM.docx]

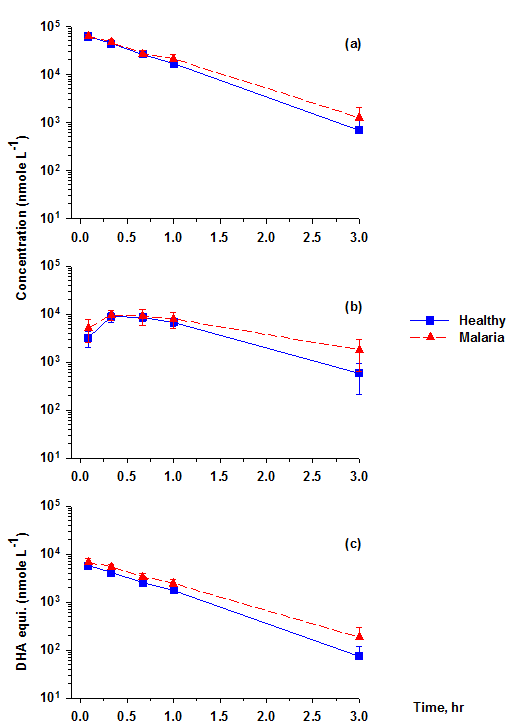


**Additional file 7**. Direct comparison of the PK/PD profiles of I.V. AL/lys between healthy self-controls (n = 10) and infected (n = 8) rhesus monkeys. a) Parent drug AL and b) its metabolite 2-OHAL profiles as measured by HPLC-ECD, and c) bioactivity profile as measured by bioassay. Values are mean + 95% CI.
